# Dual inhibition of TGFβ and AXL as a novel therapy for human colorectal adenocarcinoma with mesenchymal phenotype

**DOI:** 10.1007/s12032-021-01464-3

**Published:** 2021-02-11

**Authors:** Davide Ciardiello, Bernadette Blauensteiner, Nunzia Matrone, Valentina Belli, Thomas Mohr, Pietro Paolo Vitiello, Giulia Martini, Luca Poliero, Claudia Cardone, Stefania Napolitano, Vincenzo De Falco, Emilio Francesco Giunta, Vincenza Ciaramella, Carminia della Corte, Giusi Barra, Francesco Selvaggi, Renato Franco, Federica Zito Marino, Antonio Cuomo, Floriana Morgillo, Teresa Troiani, Maria Sibilia, Fortunato Ciardiello, Erika Martinelli

**Affiliations:** 1 Medical Oncology, Department of Precision Medicine, Università Degli Studi Della Campania Luigi Vanvitelli, Napoli, Campania Italy; 2 Department of Medicine I, Comprehensive Cancer Center, Research, Medical University of Vienna, Vienna, Austria; 3 Institute of Cancer Research, Department of Medicine I, Medical University of Vienna, Vienna, Austria; 4 General and Oncologic Surgery, Department of Medical, Surgical, Neurologic, Metabolic and Ageing Sciences, Università Degli Studi Della Campania Luigi Vanvitelli, Napoli, Campania Italy; 5 Pathology Unit, Department of Mental and Physical Health and Preventive Medicine, Università Degli Studi Della Campania Luigi Vanvitelli, Napoli, Campania Italy; 6 Gastroenterology Unit, Ospedale Umberto I, Nocera Inferiore, Italy

**Keywords:** AXL, TGFβ, Colorectal cancer, EMT

## Abstract

A subset of colorectal cancer (CRC) with a mesenchymal phenotype (CMS4) displays an aggressive disease, with an increased risk of recurrence after surgery, reduced survival, and resistance to standard treatments. It has been shown that the AXL and TGFβ signaling pathways are involved in epithelial-to-mesenchymal transition, migration, metastatic spread, and unresponsiveness to targeted therapies. However, the prognostic role of the combination of these biomarkers and the anti-tumor effect of AXL and TGFβ inhibition in CRC still has to be assessed. To evaluate the role of AXL and TGFβ as negative biomarker in CRC, we conducted an in-depth in silico analysis of CRC samples derived from the Gene Expression Omnibus. We found that AXL and TGFβ receptors are upregulated in CMS4 tumors and are correlated with an increased risk of recurrence after surgery in stage II/III CRC and a reduced overall survival. Moreover, we showed that AXL receptor is differently expressed in human CRC cell lines. Dual treatment with the TGFβ galunisertib and the AXL inhibitor, bemcentinib, significantly reduced colony formation and migration capabilities of tumor cells and displayed a strong anti-tumor activity in 3D spheroid cultures derived from patients with advanced CRC. Our work shows that AXL and TGFβ receptors identify a subgroup of CRC with a mesenchymal phenotype and correlate with poor prognosis. Dual inhibition of AXL and TGFβ could represent a novel therapeutic strategy for patients with this aggressive disease.

**Supplementary Information:**

The online version contains supplementary material available at 10.1007/s12032-021-01464-3.

## Introduction

The prognosis of localized colorectal cancer (CRC) is improved due to early detection, surgery, and adjuvant systemic treatments. However, the outcome of patients with metastatic CRC (mCRC) remains poor with a 5-year survival rate of approximately 14% [1]. Not all patients with early tumors are cured after surgical intervention, since there are some patients with aggressive disease that rapidly progress due to lack of cancer sensitivity to chemotherapies and/or targeted therapies [2]. Therefore, CRC is a heterogenous disease with a landscape of genomic alterations, which are involved not only in tumor initiation but also in disease progression and in resistance to treatments [3]. In this scenario, major efforts are needed to understand the complex molecular biology of CRC, in order to identify novel and more effective therapeutic targets and related biomarkers.

Recently, the Colorectal Cancer Subtype Consortium performed a large transcriptomic analysis and identified four different molecular subtype (CMS): CMS1 (MSI Immune, ≈14% of cases), CMS2 (Canonical, ≈37% of cases), CMS3 (Metabolic, ≈13% of the cases), and CMS4 (Mesenchymal, ≈23% of the cases) [4]. This molecular classification system can provide biological interpretability of CRC. CMS4 tumors carry the mesenchymal phenotype with a TGFβ-activated stroma and exhibit the worst relapse-free survival after surgery and the worst prognosis among the four CMS subgroups.

The TGFβ signaling pathway plays a controversial role in CRC tumorigenesis, while in early stages of cancer development, TGFβ displays a tumor suppressor activity, in metastatic CRC TGFβ favors tumor growth, invasion, EMT, metastatic spread, and immune evasion [5–9].

AXL is a member of the TAM receptor tyrosine kinase (RTK) family and regulates cell growth, migration, and angiogenesis [10, 11]. High levels of AXL expression are correlated with reduced overall survival (OS) in early-stage CRC and represent a mechanism of primary and/or secondary resistance to anti-epidermal growth factor receptor (EGFR) therapies [12, 13]. Emerging evidence suggests a functional crosstalk between the TGFβ and AXL signaling pathways in hepatocellular carcinoma (HCC) and breast cancer, while their role in CRC has never been addressed before [14, 15]. Therefore, we sought to evaluate the potential coherence between TGFβ and AXL signaling in human mCRC and examine a dual receptor inhibition approach. We showed that AXL expression is upregulated in CMS4 tumors. Moreover, high levels of AXL and TGFBR2 correlate with augmented risk of recurrence after surgery and with a reduced OS. Finally, we identify that a combined inhibition of AXL and TGFβ showed a significant anti-tumor activity in vitro and in patient-derived spheroids from primary and secondary CRC tumors. Thus, our treatment approach may represent a novel therapeutic strategy for advanced CRC.

## Materials and methods

### Dataset

Raw data of CRC samples of stage I-IV were retrieved from the Gene Expression Omnibus database with the accession number GSE40967. The CRC tumor sets were analyzed with the GPL571 platform Affymetrix Human Genome U133A 2.0 Array. The study cohort includes 750 patients with stage I to IV CRC who underwent surgery between 1987 and 2007 in seven centers. 566 of 750 (75%) samples fulfilled RNA quality requirements. Gene sequences were annotated based on the ENTREZ IDs of the NCBI platform. Gene expression data of patients who had no clinicopathological information were excluded from analysis (Supplementary Table 1).

### Background correction and normalization

Microarray analysis was carried out with R software. Data processing and normalization were performed using the robust multi-array average algorithm (RMA) as implemented in the ‘affy’ package for R/Bioconductor (available at http://www.bioconductor.org/). Data processing includes background correction, between array intensity adjustment, and transformation to a logarithm-like scale.

### Array, batch and outlier removal

CEL files were read into R using the ‘affy’ package. Quality control was performed using the arrayQualityMetrics package. Arrays marked as outliers in any of the metrics (distance between arrays, boxplots, relative log expression, normalized unscaled standard error, MA plots, and spatial distribution) were excluded from further analysis. After RMA normalization (R package ‘affy’), the resulting expression sets were subjected to a second QC analysis, as described above. Further batch effects were corrected with the’combat’ R package.

### CRC subtyping

CMS classifications were performed using the R package’CMScaller’ [16]. The prediction confidence is estimated from gene sampling and samples with a false discovery rate (FDR)-adjusted *p*-value > 0.05 were “not assigned” (NA). The classifier is dependent on gene expression values from the immune and stromal compartments of tumors. Using this algorithm, 91% of the samples were identified to be highly representative of that particular consensus molecular subtype. 9% of samples were not clearly assigned to any type, referred to as mixed subtype.

### Gene threshold

The threshold for high and low risk groups was determined by maximizing the log-rank statistics using the ‘survminer’ package of R. Briefly, samples are ranked according to gene expression and the gene expression of the sample that maximizes the log-rank statistics between the two groups is chosen as threshold. Overall, 516 CRC samples were stratified according to their gene expression levels.

### Survival analysis

Survival was estimated with the Kaplan–Meier method and described as the median or rate at specific time points followed by log-rank test in R software. A *p*-value less than 0.05 was considered to indicate a statistically significant difference.

### Correlation analysis

Pearson’s correlation coefficients between mRNA expression values were calculated. The correlograms were combined with the significance test and all entries with a *p*-value < 0.01 were removed from the plots.

### Statistical analysis

In all statistical analyses, a *p*-value of less than 0.05 or 0.01 was considered significant. Significance is presented for individual experiments (asterisks in figures). Comparisons between groups to assess statistical significance were performed with two-tailed paired Student’s *t*-tests.

### Cell lines

Human HCT116, LoVo, SW480, LIM1215, and SW48 CRC cancer cell lines were obtained from the American Type Culture Collection (ATCC) and authenticated by IRCCS “Azienda Ospedaliera Universitaria San Martino-IST Istituto Nazionale per la Ricerca sul Cancro, Genova,” (Italy). Human HCT116, LoVo, SW480, LIM1215, and SW48 CRC cancer cell lines were grown in RPMI- 1640 (Lonza), supplemented with 10% fetal bovine serum (gibco, purchased by thermofisher) and 1% penicillin/streptomycin. All cancer cells were grown in a humidified incubator with 5% of carbon dioxide and 95% air at 37 °C and were routinely screened for the presence of mycoplasma (Mycoplasma Detection Kit; Roche Diagnostics).

### Drugs

The TGFBR1 inhibitor, galunisertib (LY2157299), and the AXL inhibitor, bemcentinib (R428), were purchased by Selleckchem. Drugs were dissolved in sterile dimethylsulfoxide (DMSO) at a 10 mM stock solution concentration and stored in aliquots at − 20 °C.

### Western blot analysis

Protein lysates were obtained by homogenization in RIPA lysis buffer (Sigma-Aldrich, MO, USA) supplemented with protease and phosphatase inhibitors (Hoffmann-La Roche). Protein extracts were quantified with the Bradford assay (Bio-Rad, CA, USA) and equal amounts of total protein were separated by a 4–15% gradient mini pre-cast TGX gel (Bio-Rad). Proteins were transferred onto a nitrocellulose membrane, blocked with BSA solution and incubated with primary antibodies (Cell Signaling) overnight on 4 °C. The secondary antibody was incubated at RT for 1 h before detection. Immunocomplexes were detected with the enhanced chemiluminescence kit ECL plus, by Thermo Fisher Scientific (Rockford, IL) using the ChemiDoc device (Bio-Rad). Each experiment was performed in duplicate.

### RNA isolation and qRT-PCR

The mRNA levels of AXL, TGFBR1, and TGFBR2 were measured by real-time quantitative polymerase chain reaction (RT-qPCR). Total RNA was isolated using TRIzol reagent (Life Technologies) and was reversely transcribed into cDNA using SensiFast reverse transcriptase (Bioline) according to the manufacturer instruction. Amplification was conducted using the SYBER Green PCR Master Mix (Applied Biosystems). The quantified value of each sample was normalized to *18S* expression in the same sample, which was amplified simultaneously with the target genes. The relative gene expression was quantified using the 2^−∆∆*t*^ method. Each sample was tested in duplicate using a Quant studio 7 Flex (Applied Biosystem).

### Migration

Chamber of transwell (6.5 mm diameter, 8 μm pore size polycarbonate membrane, Corning) was used to evaluate the migratory capacity of HCT116 and LoVo cells with or without inhibitor treatment, i.e., galunisertib, bemcentinib and the combination of the two drugs. A cell concentration of 25 × 10^4^ cells in 200 μl medium without FBS was added to each migration (upper) chamber of the transwells. Chemotaxis was induced by addition of 10% fetal bovine serum to the medium in the lower chamber. Cells were allowed to migrate from the upper compartment through the membrane towards the lower compartment along the chemo-attractant gradient. After incubation for 48 h, non-migrating cells were removed with cotton swabs, and the cells that migrated into the lower surface of the filters were stained with 0.1% crystal violet, solubilized with 1% isopropanol-HCL, and quantified by measuring the absorbance at 560 nm. Each experiment was performed in duplicate and repeated at least twice.

### Colony-forming assay

Colony-forming assay was performed to evaluate the long-term proliferative potential in HCT116 and LoVo cells treated with galunisertib, bemcentinib, and the combination of the two drugs. Cells were seeded on 6-well tissue culture dishes at 1000–3000 cells/well and treated with the indicated drug/s at different doses. After 96 h, the medium containing the drug/s was removed and cells were maintained for 10 days and fresh culture media was replaced every 3 days. Cells were stained with 0.1% crystal violet solubilized with 1% isopropanol-HCL and quantified by measuring the absorbance at 560 nm. Treatment conditions were normalized to untreated cells, respectively. Each experiment was performed in triplicate and repeated at least twice.

### Generation of three-dimensional (3D) ex vivo cultures, as spheroids and drug treatment

Fresh tissue biopsies from 13 primary tumors or liver metastasis from patients with CRC were used to establish 3D tumor cultures. The protocol has been approved by the local Ethics Committee and all patients gave their written informed consent to the use of the tumor sample. Tumor tissues were weighed, washed, and cut in fragments. Briefly, tumor fragments were incubated with digestion medium (DMEM F-12, Sigma-Aldrich) supplemented with 2% penicillin/streptomycin 10X amphotericin, 2X collagenase and Hyaluronidase for up 16 h on a 37 °C under agitation. Undigested fragments and debris were filtered through a 100 µm cell strainer (BD-Falcon) followed by centrifugation for 5 min at 1200 rpm. The supernatant was removed, and the pellet was further re-suspended in an ice-cold 1:1 mixture of growth medium and Matrigel (BD-Falcon) and seeded in 24-well plates (Corning) for cytotoxicity experiments. The Matrigel droplets were polymerized for 10 min at 37 °C and growth medium was added. The 3D spheroids were treated with 1 µM of bemcentinib and/or 10 µM galunisertib for 14 days. The cell viability was assessed by 3-(4,5-dimethylthiazol-2-yl)-2,5-di-phenyltetrazoliumbromide (MTT-Sigma-Aldrich) assay. Matrigel was degraded using Cell Recovery Solution (BD-Falcon), according to the manufacturer’s protocol. Retrieved, 3D spheroids were centrifuged as described above and lysated and the absorbance at 590 nm was detected using a spectrophotometer. Untreated spheroids were defined as 100% viable.

### FoundationOne analysis

To evaluate the genomic landscape of the 3D patient-derived spheroid cultures, we analyzed tumor formalin-fixed paraffin embedded (FFPE) with the FoundationOne assay. The test required 10 blank slides to provide at least 55 ng of genomic DNA to ensure enough DNA for quality control. The assay detects alterations in a total of 324 genes including the assessment of the microsatellite instability (MSI) status and tumor mutational burden (TMB). Using the Illumina®HiSeq 4000 platform, hybrid-capture–selected libraries are sequenced to high uniform depth (targeting > 500X median coverage with > 99% of exons at coverage > 100X). FoundationOne®CDx Full Specification Information; https://www.accessdata.fda.gov/cdrh_docs/pdf17/P170019C.pdf

## Results

### AXL and TGFBR2 are associated with a mesenchymal CRC subtype

**Fig. 1:**
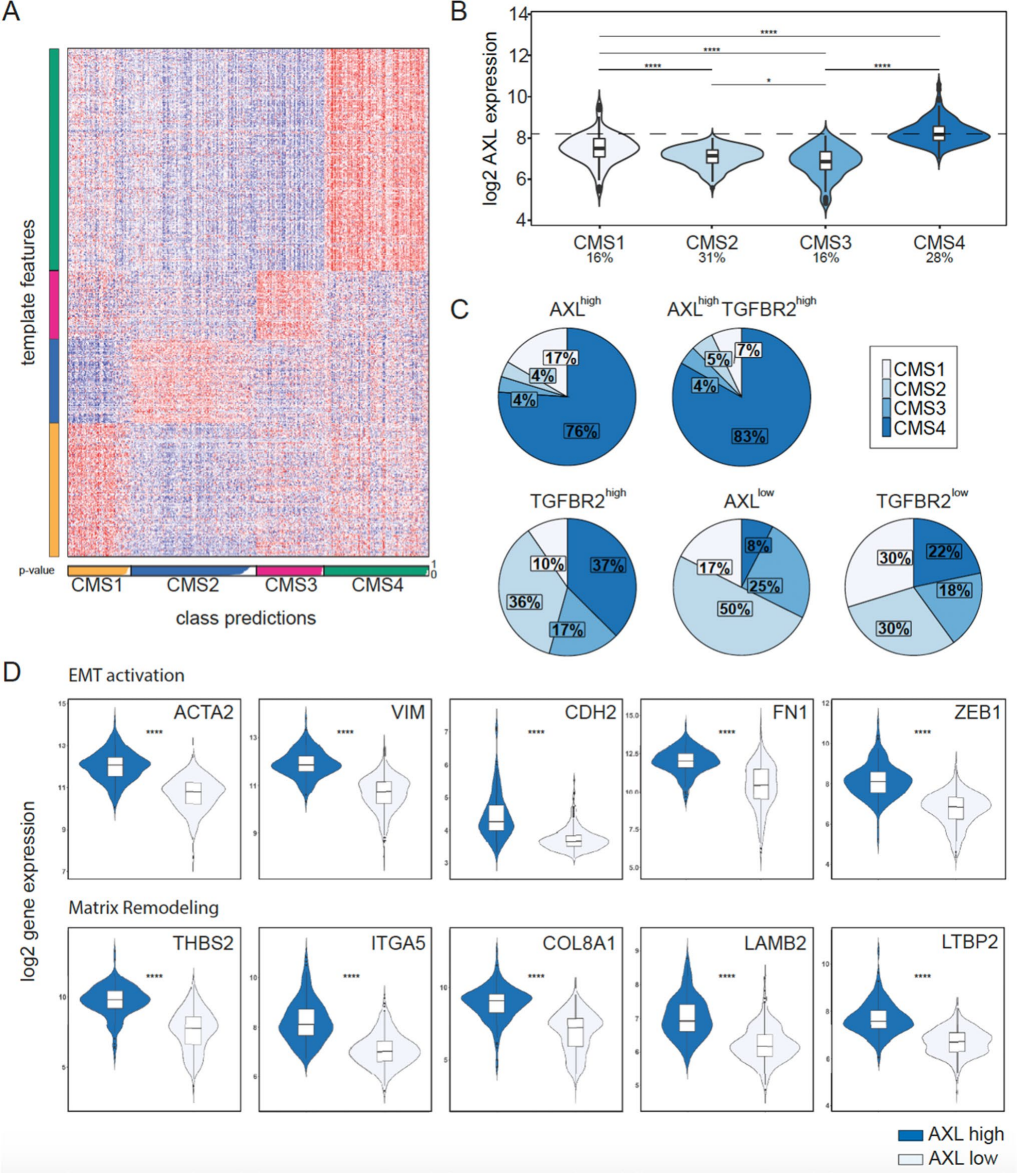
AXL and TGFBR2 are associated with CRC progression. **a** Heatmap of assigned CMS subtypes to tumor samples based on template features by the CMS caller algorithm. The subtypes are highlighted in yellow (CMS1), blue (CMS2), pink (CMS3), and green (CMS4). The bars are not filled due to non-matched samples. The *p*-value is given from 0 to 1. **b** Violin and box plot of AXL expression in the CMS subtypes. The percentages below indicate the relative number of samples that were assigned to the individual CMS subtypes, respectively. **c** Pie charts of the CMS subtype distributions within the gene/s high versus gene/s low group. The cut-off of AXL and TGFBR2 was analyzed with maximum log-rank statistics. Rounding errors may cause little deviations from 100%. **d** Violin and box plots of several genes associated with epithelial-to-mesenchymal transition and matrix remodeling in AXL high compared to AXL low. Significance: *p*-value > 0.05 ns, < 0.05*, < 0.01**, < 0.001***, < 0.0001****

CRC is a heterogenous disease with molecular phenotypes that can influence the patient prognosis and survival [4]. In order to analyze the molecular classification potential of AXL and TGFBR2 to distinguish low- and high-risk CRC patients, a publicly available dataset from the GEO database was analyzed. From the 750 CRC samples, a total of 516 tumor samples fulfilled RNA quality and remained for downstream analysis after quality control and normalization. These samples were subjected to the CMScaller algorithm [16]. In total, 516 CRC samples were assigned to one of the CMS subtypes, accordingly (Fig. 1a). CMS1 (MSI) and CMS3 (high KRAS mutations) contribute to 16% of all tumor subtypes, respectively. Approximately, 30% of all cancer subtypes fall into the category CMS2 (elevated EGFR levels) or CMS4 (mesenchymal-like). The latter one displayed upregulation of genes, involved in EMT, matrix remodeling, TGFβ signaling, angiogenesis, and inflammatory-related systems. AXL expression was found to be significantly distinct in all subtypes, being highest expressed in the CMS4 subtype (Fig. 1b, *p* < 0.0001). Quantitative changes could also be seen in terms of subtype distributions between high and low gene expressing groups (Fig. 1c). Therefore, tumor samples were classified into groups according to whether they fall into the low or high gene expression group by using maximum log-rank statistics and the survminer package. High levels of AXL and TGFBR2 were observed in 83% of CMS4 tumors suggesting that dual analysis of AXL and TGFBR2 could contribute to better identify CRC with a mesenchymal phenotype.

Of note, while CMS4 is enriched in both the AXL^high^ and TGFBR2^high^ groups over the respective low expressing groups, high levels were even more pronounced for AXL over TGFBR2. Similarly, upregulation of TGFBR1 and AXL is strongly correlated with CMS4 tumors (Supplementary Figure 1). Thus, AXL could represent a novel molecular marker for advanced staged mesenchymal-like colon tumors. Indeed, genes associated with EMT, matrix remodeling, and cellular adhesion were significantly upregulated in AXL^high^ over AXL^low^ tumors samples (Fig. 1d, Supplementary Figure 2). While AXL expression strongly correlates with the expression of common EMT-like genes, such as vimentin, N-Cadherin, and fibronectin, it shows only minor or even negative correlation with epithelial markers. Interestingly, AXL was strongly correlated with high levels of TGFβ (Supplementary Figure 2).

### The AXL/TGFBR2 gene signature improves the prediction of early-stage CRC and is associated with a poor overall survival

**Fig. 2:**
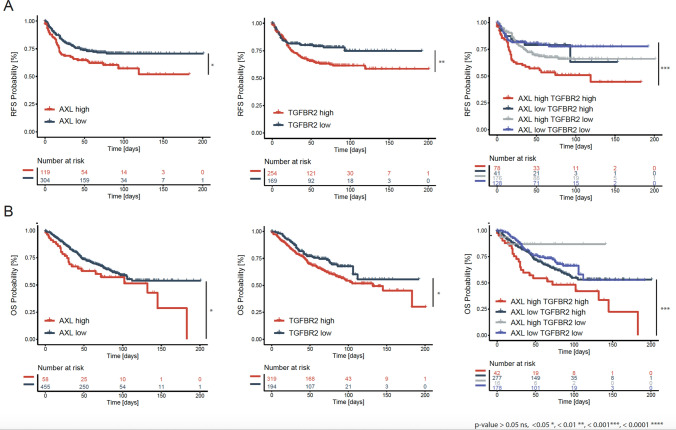
Kaplan–Meier survival analysis in CRC based on AXL and TGFBR2 expression. **a** The relapse-free survival (RFS) of CRC patients with low and high AXL and/or TGFBR2 expression in tumor tissue. **b** The overall survival (OS) of CRC patients with low and high AXL and/or TGFBR2 expression in tumor tissue. Variables: Time in days (survival or censoring time), number at risk, gene (gene low or highly expressed). Single-gene survival plots: low (black), high (red); gene signature plot: low/low (blue), low/high (shades of gray), high/high (red). Significance: *p*-value > 0.05 ns, < 0.05*, < 0.01**, < 0.001***, < 0.0001****

To identify the prognostic value of AXL, we determined differential changes in gene expression of the tumor samples assigned to either the AXL^low^ or the AXL^high^ group. The relapse-free survival (RFS) of CRC patients with AXL^high^ expression in the tumor tissue (*n* = 167) was significantly lower than that for AXL^low^ patients (*n* = 341). Patients in the TGFBR2^high^ group (*n* = 315) also showed a worse prognosis as compared to the low expressing group (*n* = 193). Dual upregulation of AXL and TGFBR2 correlated with a significantly increased risk of relapse (*p* = 0.00043) (Fig. 2a). Similarly, patients with AXL^high^ and TGFBR2^high^ tumors had a reduced overall survival (OS) compared with AXL and TGFBR2 low expressing tumors (*p* = 0.0064) (Fig. 2b). However due to a small number of patients in same subgroups, there was a statistical imbalance that render very difficult to extrapolate any conclusion.

### Effects of dual AXL and TGFβ blockade in human CRC cell lines

**Fig. 3:**
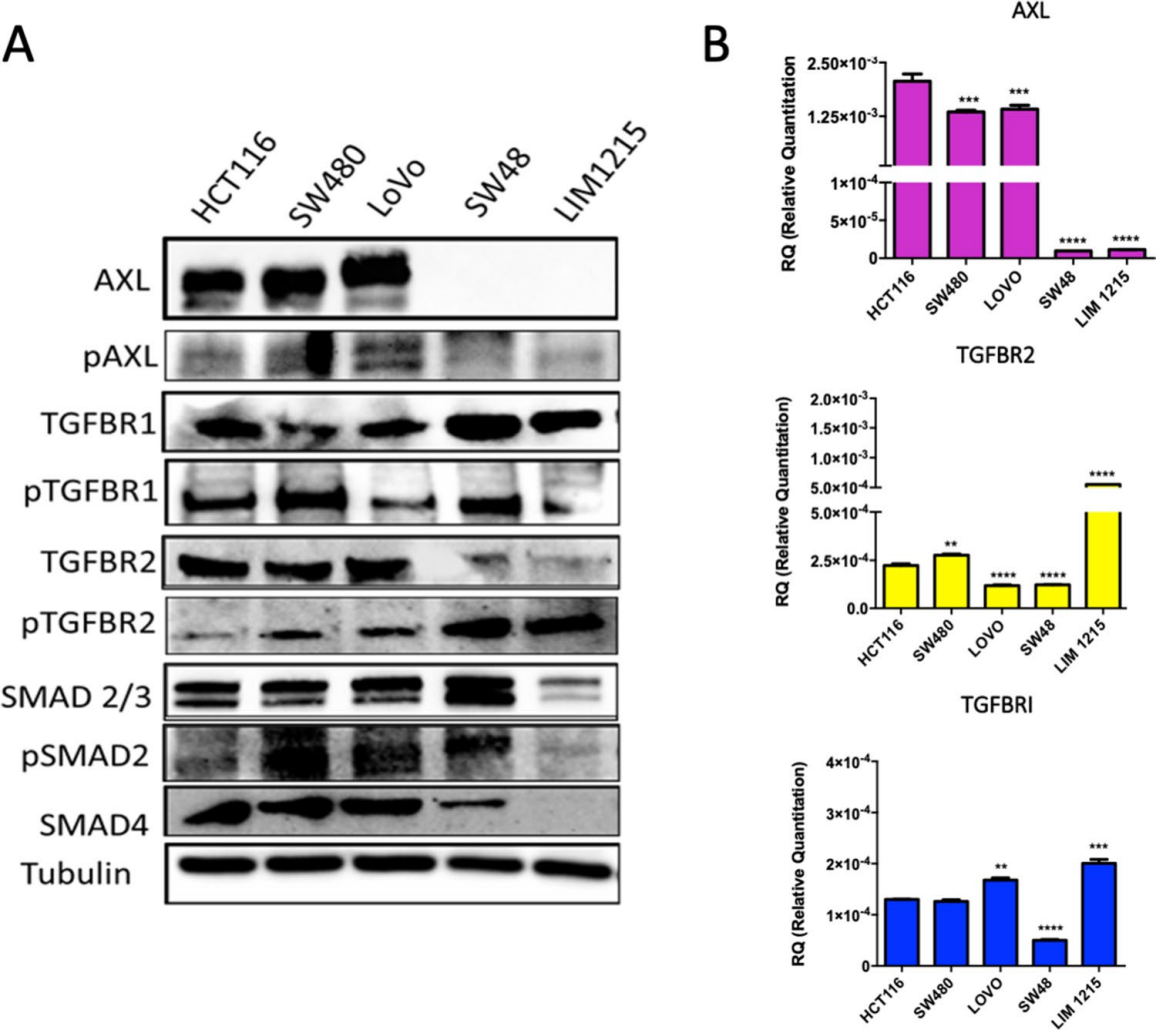
AXL and TGF-β expression and functional inhibition in a panel of CRC cell lines. **a** Western blot analysis of AXL and TGFβ receptor expression in human CRC cell lines. AXL is expressed in HCT116, SW480, and LoVo cells. **b** Relative gene expression of AXL, TGFBR2 and TGFBR1 in a panel of CRC cell lines

We studied the anti-tumor activity of AXL and TGFβ blockade in in vitro models of human CRC. Therefore, AXL and TGFβ receptors expression was assessed on mRNA and protein level in a panel of human CRC cell lines (HCT116, SW480, LoVo, SW48, LIM1215) including three RAS-mutant (RASm) (HCT116, SW480, and LoVo) and two RAS wild-type (RASwt) (SW48, LIM1215) cancer cell lines. While TGFBR1 and TGFBR2 were expressed heterogeneously by all cell lines (Fig. 3a, b), AXL was only expressed in the RASm cell lines. Thus, RASm cell lines show the highest AXL protein expression and TGFβ signaling activation, making them suitable for the study of the dual receptor blockade.

**Fig. 4:**
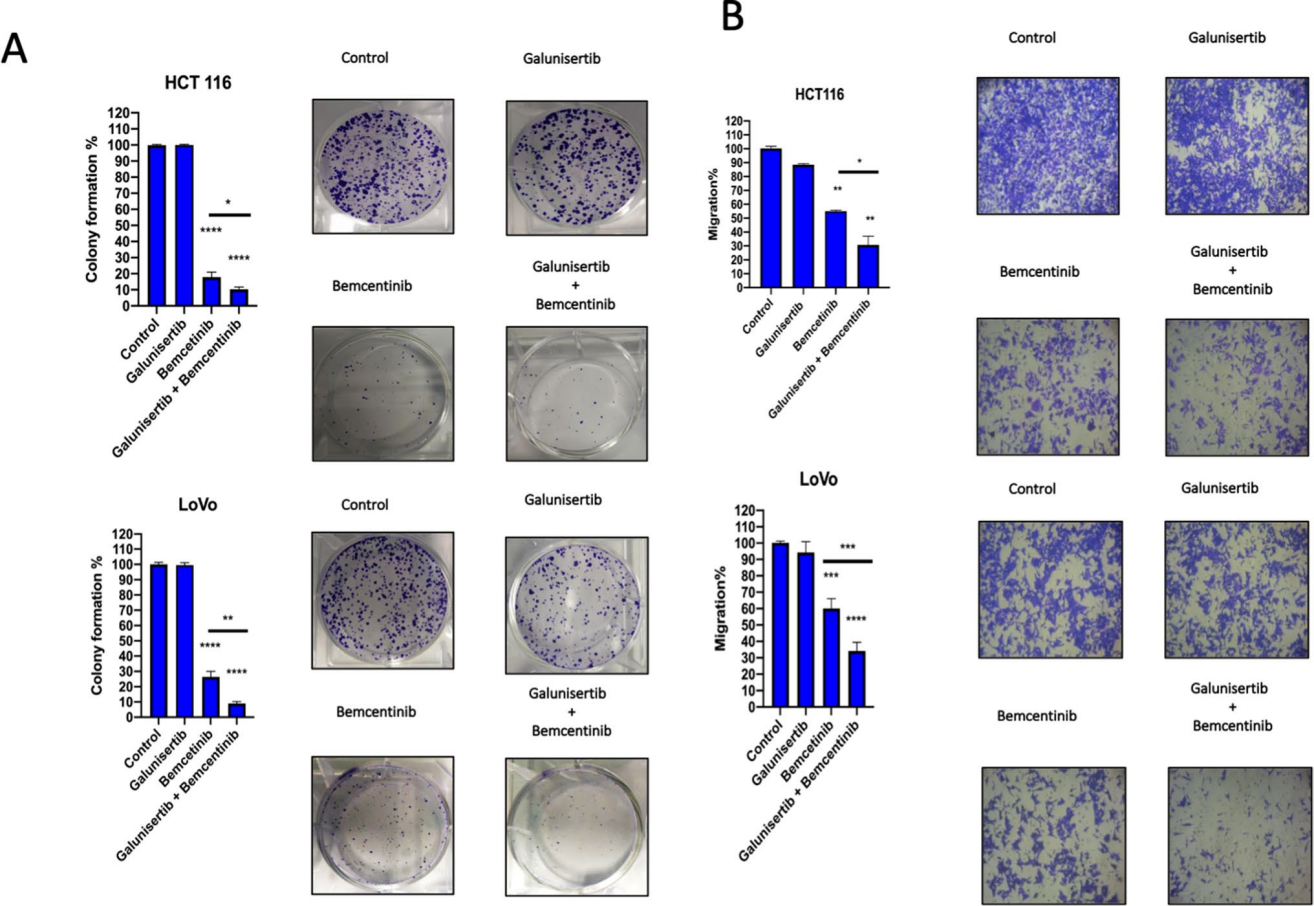
Colony-forming and migration assay in HCT116 and LoVo cells. **a** Colony-forming assay of HCT116 and LoVo cells treated with galunisertib 10 µM and/or bemcentinib 1 µM. Error bars indicate the standard deviation compared to single treatment. *T*-student test was used for statistical analysis, *p*-value < 0.05*, < 0.01**, < 0.001***, < 0.0001****. **b** Transwell migration assay at 48 h of LoVo cells treated with galunisertib 10 µM and/or bemcentinib 1 µM. Each assay was performed in duplicate. *T*-student test was used for statistical analysis, *p*-value < 0.05*, < 0.01**, < 0.001***, < 0.0001****

To assess the potential anti-tumor activity of dual inhibition of AXL and TGFβ receptors we performed a colony-forming and migration assay in HCT116 and LoVo cells (Fig. 4a, b). In line with literature, HCT116 and the LoVo cell lines are classified, respectively, into CMS4 and CMS1 subgroups [17]. Despite being classified as CMS1, the LoVo cell line displays a mesenchymal phenotype with an upregulation of AXL and EMT markers expression, rendering them a good model to assess the effect of AXL and TGFβ blockade [17].

For colony-forming assay cancer cells were seeded in multi-well plates at clonal density and treated with the TGFBR1 inhibitor galunisertib and/or the AXL inhibitor. Galunisertib displayed marginal anti-proliferative activity, while AXL inhibition significantly reduced colony formation as compared to untreated cells (Fig. 4a). The addition of TGFBR1 inhibition to the AXL blockade slightly increased the inhibitory activity of AXL in HCT116 and LoVo cells by reducing colony formation. Furthermore, we evaluated the effect of AXL and TGFBR1 blockade on the invasion capability. Single-agent treatment with galunisertib displayed a modest effect on cell migration, whereas bemcentinib induced a reduction in cell migration of approximately 20–30% (Fig. 4b). Interestingly, the combination of the two drugs significantly reduced the invasiveness in HCT116 and LoVo cells as compared to single-agent treatment.

### Efficacy of dual blockade of AXL and TGFβ in ex vivo models

**Fig. 5:**
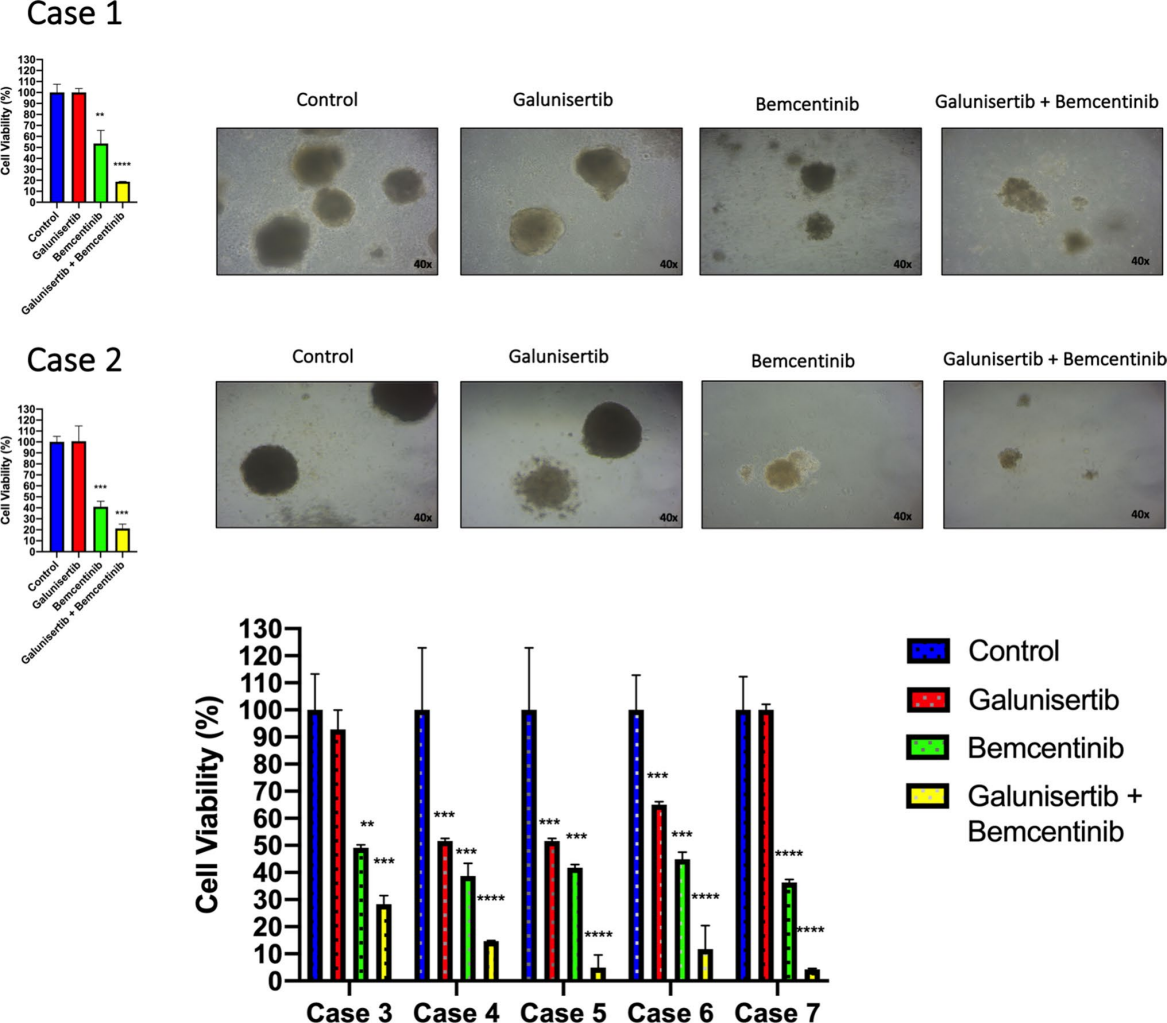
Anti-tumor activity of TGFBR1 and AXL is blocked in 3D patient-derived spheroid culture. MTT proliferation assay for colorectal cancer spheroids of case 1–7 treated with 10 µM galunisertib and 1 µM bemcentinib. Graphical representation of representative images with a magnification of 40×. *T*-student test was used for statistical analysis for comparing the treatments with the untreated control, *p*-value < 0.05*, < 0.01**, < 0.001***, < 0.0001****

Emerging evidence suggests that CRC patient-derived spheroid cultures may retain the characteristics of the primary tumors and that they could represent a compelling tool for investigating the sensitivity of cancer cells to anti-cancer treatments [18, 19]. In this regard, samples from 13 CRC patients that received surgery for the removal of the primary tumor or a liver biopsy of a metastasis were collected and cultured as ex vivo 3D spheroid cultures to study the effects of AXL and TGFβ inhibition in a more clinical setting. In total, 7 out of 13 spheroid cultures were established with a successful rate of 53. Patient characteristics for the seven successfully established spheroids are summarized in Table 1. To assess the response to galunisertib, bemcentinib, and/or to the combination of the drugs, 3D spheroid cultures were treated for 14 days with the respective drugs and cancer cell proliferation was evaluated by the MTT assay. In all the seven cases, treatment with bemcentinib revealed a significant cell growth inhibition of about 40–50% as compared to untreated controls. On the other hand, treatment with the single-agent galunisertib determined cancer cell growth inhibition in 3 out of 7 spheroid cultures (cases 4, 5 and 6). However, the dual blockade of AXL and TGFBRI led to almost complete cell growth inhibition of up to 90% (Fig. 5).

**Table 1:** Patients characteristics of 3D spheroid cultures

Case	Sex	Age	Stage	Biopsy site	Molecular alterations
1	M	57	IV	COLON	KRAS G12V MSS BCORL1 L1326FS*38 CHECK 1R160H
2	M	55	IV	LIVER	MSI AXL T343M PTCH1 L39fs*41 PIK3CA H1047R PIK3R1 D68fs*7, N257fs*10 CTNNB1 T41A
3	M	72	IV	COLON	BRAF V600E MSS NSD3 amplification ZNF3 amplification
4	F	75	IV	COLON	KRAS G13D MSS PIK3CA H1047L SMAD4 R361C, P522fs*4 FANCC splice site 522-1G > C MED12 G44S
5	F	76	II	COLON	KRAS G12D MSI ATM K1820fs GATA 3 W112fs*83, s237fs*67
6	F	53	III	COLON	MSS MYC amplification PIK3CA T1025I
7	F	60	IV	COLON	MSS KRAS G12V CHEK2 loss exons 3–7 APC E941*, E1494fs*12 TP53 H179Y

The table summarizes the main clinical patient information of whom 3D spheroid cultures were established. Primary colon tumors were isolated from 6 out of 7 patients (case 1, 3–7), and a liver biopsy was obtained from one patient (case 2). The molecular characterization of tumor samples was performed with the FoundationOne test

*MSI* Microsatellite instability; *MSS* Microsatellite stable

To better understand the different treatment responses, the tumor samples were analyzed by extended next-generation sequencing (NGS) analysis with FoundationOne analysis. Interestingly, one patient (case 2) showed a missense alteration in the AXL extracellular domain (T343M) of uncertain significance. Another patient (case 4) displayed a SMAD4 R361C mutation that determines a loss of function in the protein, impairing the binding with Smad2/3 favoring the shift from the anti-tumoral to the pro-tumoral activity of TGFβ [20]. Thus, in this case, the blockade of TGFβ signaling could determine an anti-proliferative activity.

## Discussion

CRC is a heterogenous disease with a complex landscape of genetic alterations that influence the disease clinical behavior and evolution and that affect susceptibility to anti-cancer treatments. During the last twenty years, major efforts have been made to identify key molecular drivers of CRC in order to offer a personalized and molecularly driven treatment for each patient. Some major clinical results have been obtained following the discovery that certain gene alterations, such as activating KRAS or NRAS point mutations, BRAFV600E mutation, HER2 gene amplification or the MSI high (MSI-H) status may characterize specific subgroups of patients that could be treated with selective anti-cancer therapies [21, 22]. Alongside the identification of single-gene alterations, the CMS classification allows to stratify CRC according to distinct molecular subgroups, which are based on a complex gene signature analysis [4]. Noteworthy, CMS4 (mesenchymal) tumors are associated with prominent TGFβ activation, stromal invasion, and angiogenesis and have a higher risk of recurrence after surgery with reduced OS as compared to CMS2 or CMS3 patients [4]. Disruption of TGFβ signaling plays a pivotal role in CRC pathogenesis as it causes EMT in cancer cells, resulting in an aggressive phenotype [23]. Indeed, in silico analysis of human CRC tumors shows a poor prognosis of TGFBR2^high^ patients.

Emerging evidence suggests that AXL overexpression could be associated with an aggressive disease that correlates with reduced survival in early-stage CRC and with resistance to anti-EGFR treatments [12, 13]. In this regard, our group has previously demonstrated that the blocking of AXL with foretinib exhibits anti-tumor activity since it reduces cancer cell survival and migration capability in vitro and determines tumor regression in vivo [11]. Furthermore, we identified AXL as a prognostic marker in therapy-resistant metastatic CRC (13). As these findings indicated, upregulation of AXL is involved in CRC development and patient’s prognosis.

A possible connection of TGFB and AXL was recently described in HCC. AXL can dysregulate TGFβ signaling through the aberrant phosphorylation of the Smad3 linker region, which causes the repression of the anti-proliferative activity and promotes EMT [14]. Goyette and colleagues had also shown that AXL is necessary for inducing TGFβ-mediated EMT, migration, and metastatic spread which confers resistance to anti-HER treatments in a breast cancer model [15]. Yet, a possible context of both receptors in CRC has not been shown. In order to further investigate the correlation between AXL and TGFβ, we have conducted an in silico analysis of the publicly available dataset GSE40967, including stage I–IV CRC patients with clinical annotation such as RFS and OS [24]. AXL was upregulated in 76% of patients with colorectal cancer and strongly correlated with a mesenchymal phenotype, whereas 37% of TGFBR2^high^ and 65% of tumors TGFBR1^high^ were attributed to the CMS4 subtype. Upregulation of AXL and TGFBR1 or TGFBR2 was observed in approximately 80–90% of CMS4 samples, suggesting that dual analysis of these biomarkers could be a better strategy to identify tumors with these mesenchymal characteristics. The optimal therapeutic strategy after surgery, including the choice of chemotherapy and the duration of the treatments, is still debated [25]. Recently, the analyses of the immune infiltrate (immunoscore) and of the circulating tumor-derived DNA have been proposed as possible biomarkers for the identification of patients with CRC at higher risk of recurrence after surgery [26–28]. Here, we provide evidence that tumors with high levels of AXL or TGFBR2 are associated with worse RFS and OS as compared to the low expressing tumors. Moreover, upregulation of both receptors was correlated with a significant increased risk of tumor relapse (*p* = 0.00043).

Collectively, these finding support the concept that enhanced dual expression of both AXL and TGFBR2 correlates with a more aggressive CRC and is associated with poor survival. Thus, there is a strong rationale for exploring the potential therapeutic efficacy of the combined inhibition of the two receptors. Robust preclinical evidence has demonstrated that dual TGFβ and PD-L1 blockade can trigger the activation of the immune system and could exhibit strong anti-tumor response in a mouse tumor experimental model [9]. Despite these promising preclinical results, the combination of TGFβ and PD-L1 inhibition demonstrated little activity in pre-treated mCRC patients [29]. Similarly, the combination of TGFBR1 blockade (PF-03446962) with the multi-kinase inhibitor regorafenib failed to obtain clinical activity in refractory mCRC [30]. Therefore, new combinatory strategies are required for targeting TGFβ in CRC. Here, we have provided experimental evidence that dual AXL and TGFβ blockade elicit a strong anti-tumor activity in a preclinical and *ex vivo* human CRC model. To support this hypothesis, we evaluated a dual treatment approach with the combination of bemcentinib and galunisertib. Indeed, the combinatory therapy showed a significant reduction in colony formation and migration as compared with single-agent treatments in CRC cell lines that express high levels of AXL and TGFβ. To further expand these preclinical observations, we obtained six primary 3D spheroid cultures from CRC patients. The combined treatment with bemcentinib and galunisertib led to a strong reduction in cancer cell viability (80–90%) in all cultures. Contrary, galunisertib alone showed an activity in only 3/7 cases and limited activity in the other spheroids. To better understand this pattern of response, we performed extended NGS gene mutation analysis on these tumor samples. Interestingly, one patient-derived spheroid that showed a potent response to TGFβ inhibition displayed a pathogenic alteration in Smad4 (R361C). This mutation has been shown to prevent its binding to Smad2/3 and thereby impair the anti-proliferative activity of TGFβ [20]. Moreover, another patient (case 2) exhibited a rare missense alteration in the AXL extracellular domain (T343M) that has been described in breast cancer [31]. The pathogenic role of this mutation is still uncertain. To date, this is the first time that AXL T343M has been reported in colorectal cancer.

With the limitations of a relatively small sample size of the primary 3D spheroid cultures, the current study represents a proof of concept that combined anti-AXL and anti-TGFβ treatments might represent a therapeutic option for selected molecular subgroups of CRC that deserves further investigation.

## Conclusion

CRC is a complex disease, with a landscape of molecular alterations that influence not only tumor initiation, but also cancer progression, invasiveness, and resistance to treatments. Here, we show that high levels of AXL and TGFBR2 correlate with the mesenchymal CMS4 subtype and reduced RFS and OS. Moreover, in a preclinical model of CRC, a functional crosstalk between AXL and TGFβ was observed. The combinatory inhibition of the two receptors showed an encouraging anti-tumor activity. Therefore, our data suggest that a subset of CRC tumors with poor prognosis depends on AXL and TGFβ signaling and thus targeted treatment could represent a promising innovative therapeutic strategy for these patients.

## Supplementary Information

Below is the link to the electronic supplementary material.

Supplementary Figure 1 AXL and TGFBR1 associate with the mesenchymal CMS4 subtype. Pie charts of the CMS subtype distributions within the gene/s high versus gene/s low group. The cut-off of AXL and TGFBR1 was determined with maximum log-rank statistics by the survminer package. Rounding errors may cause little deviations from 100%. Supplementary file1 (PDF 420 KB)

Supplementary Figure 2 AXL correlates with a cancer-related gene signature. Correlation matrix of AXL gene expression with gene signatures of EMT activation and organization, cell adhesion, epithelial cell differentiation, and TGFβ activation (from left to right, increasing grey scale intensity). Positive correlation coefficients are displayed in blue and negative correlations in red color. Color intensity and the size of the circle are proportional to the correlation coefficients. Correlations with p-value > 0.01 are considered as insignificant and removed from the plot (empty areas). Supplementary file2 (PDF 518 KB)

Supplementary Table 1. Patient and tumor characteristics of the discovery dataset. The left column gives the clinical and pathological information of the whole population of GSE40967 Discovery Dataset after data processing (n=516). The right column shows tumor and patient characteristics of a subgroup of patients, i.e. stage II/III CRC population, which was used for the analysis of the relapse free survival. Tumor and pathological characteristics are comparable between the subsets for downstream analysis. Supplementary file3 (PDF 59 KB)

## Data Availability

The dataset analyzed in the current study is the Gene Expression Omnibus database with the accession number GSE40967 (Marisa L, de Reyniès A, Duval A, et al. Gene expression classification of colon cancer into molecular subtypes: characterization, validation, and prognostic value. *PLoS Med*. 2013;10(5):e1001453. https://doi.org/10.1371/journal.pmed.1001453).
