# Supplementary material for: Dual inhibition of TGFβ and AXL as a novel therapy for human colorectal adenocarcinoma with mesenchymal phenotype
Source: Med Oncol. 2021 Feb 11;38(3):24. doi: 10.1007/s12032-021-01464-3 (PMC7878213; doi:10.1007/s12032-021-01464-3)
Supplement: Supplementary file 1 — Supplementary Figure 1 AXL and TGFBR1 associate with the mesenchymal CMS4 subtype. Pie charts of the CMS subtype distributions within the gene/s high versus gene/s low group. The cut-off of AXL and TGFBR1 was determined with maximum log-rank statistics by the survminer package. Rounding errors may cause little deviations from 100%. Supplementary file1 (PDF 420 KB) [file 12032_2021_1464_MOESM1_ESM.pdf]

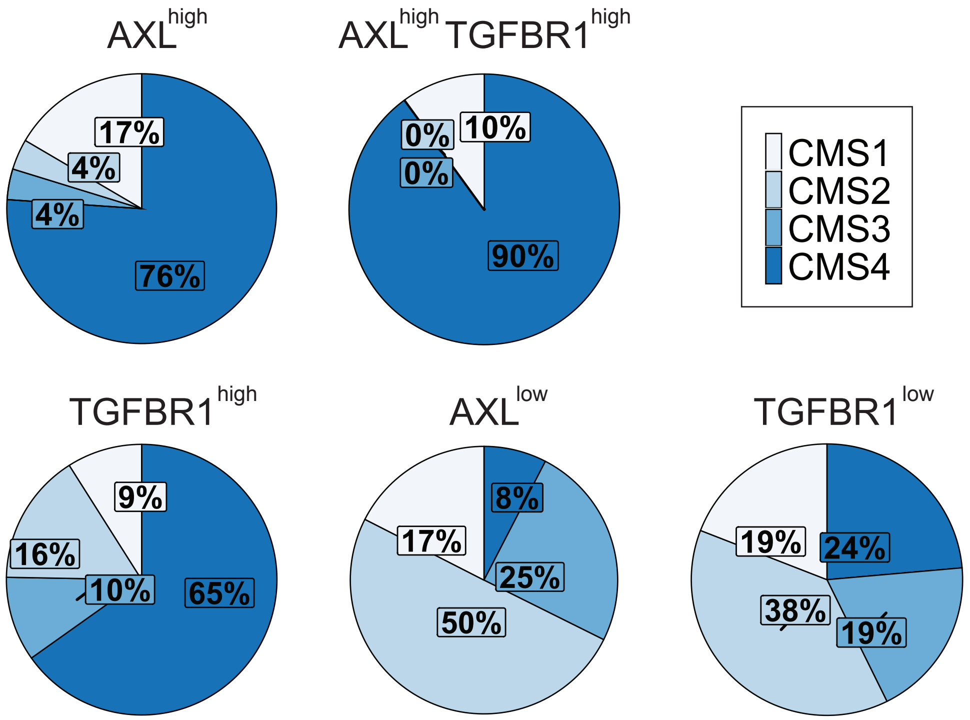
AXL^high
17%
4%
4%
76%
AXL^high TGFBR1^high
0%
10%
0%
90%
CMS1
CMS2
CMS3
CMS4
TGFBR1^high
9%
16%
10%
65%
AXL^low
8%
17%
25%
50%
TGFBR1^low
19%
24%
38%
19%
