# Supplementary material for: Dual inhibition of TGFβ and AXL as a novel therapy for human colorectal adenocarcinoma with mesenchymal phenotype
Source: Med Oncol. 2021 Feb 11;38(3):24. doi: 10.1007/s12032-021-01464-3 (PMC7878213; doi:10.1007/s12032-021-01464-3)
Supplement: Supplementary file 2 — Supplementary Figure 2 AXL correlates with a cancer-related gene signature. Correlation matrix of AXL gene expression with gene signatures of EMT activation and organization, cell adhesion, epithelial cell differentiation, and TGFβ activation (from left to right, increasing grey scale intensity). Positive correlation coefficients are displayed in blue and negative correlations in red color. Color intensity and the size of the circle are proportional to the correlation coefficients. Correlations with p-value > 0.01 are considered as insignificant and removed from the plot (empty areas). Supplementary file2 (PDF 518 KB) [file 12032_2021_1464_MOESM2_ESM.pdf]

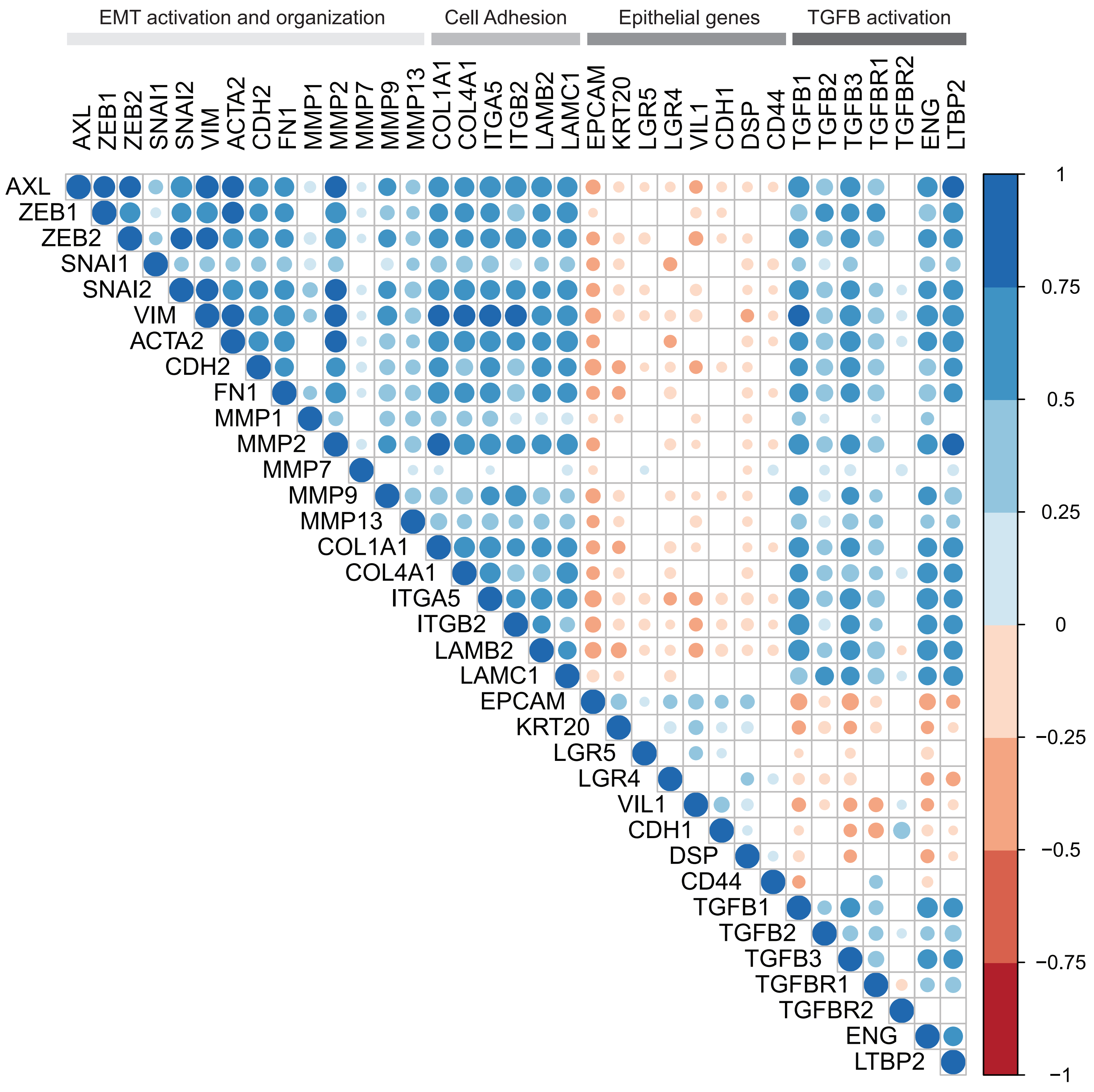
EMT activation and organization
Cell Adhesion
Epithelial genes
TGFB activation
AXL
ZEB1
ZEB2
SNAI1
SNAI2
VIM
ACTA2
CDH2
FN1
MMP1
MMP2
MMP7
MMP9
MMP13
COL1A1
COL4A1
ITGA5
ITGB2
LAMB2
LAMC1
EPCAM
KRT20
LGR5
LGR4
VIL1
CDH1
DSP
CD44
TGFB1
TGFB2
TGFB3
TGFBR1
TGFBR2
ENG
LTBP2
1
0.75
0.5
0.25
0
−0.25
−0.5
−0.75
−1
