# Supplementary material for: Dual inhibition of TGFβ and AXL as a novel therapy for human colorectal adenocarcinoma with mesenchymal phenotype
Source: Med Oncol. 2021 Feb 11;38(3):24. doi: 10.1007/s12032-021-01464-3 (PMC7878213; doi:10.1007/s12032-021-01464-3)
Supplement: Supplementary file 3 — Supplementary Table 1. Patient and tumor characteristics of the discovery dataset. The left column gives the clinical and pathological information of the whole population of GSE40967 Discovery Dataset after data processing (n=516). The right column shows tumor and patient characteristics of a subgroup of patients, i.e. stage II/III CRC population, which was used for the analysis of the relapse free survival. Tumor and pathological characteristics are comparable between the subsets for downstream analysis. Supplementary file3 (PDF 59 KB) [file 12032_2021_1464_MOESM3_ESM.pdf]

Characteristics	GSE40967 Discovery Dataset	
**Samples**	**General population n = 516**	**Stage II/III population n = 430**
**Age [year]**		
Mean age	67	68
Age range	22-97	22-97
**Sex total (in %)**		
female	237 (46)	193 (45)
male	279 (54)	237 (55)
**TNM stage total (in %)**		
I	31 (6)	0
II	238 (46)	238 (55)
III	192 (37)	192 (45)
IV	53 (10)	0
**Location (%)**		
proximal	207 (40)	178 (41)
distal	309 (60)	252 (59)
**Adj. Chemotherapy (%)**		
Yes	211 (41)	186 (43)
No	289 (56)	242 (56)
**Relapse [months]**		
Median follow-up	46.0	50.0
Follow-up range	0-201	0-201
**Relapse events (%)**		
Yes	157 (40)	122 (28)
No	351 (68)	301 (70)
**Median OS (months)**		
Yes	166 (32)	127 (30)
No	347 (67)	301 (58)
**CMS prediction (%)**		
CMS1	82 (16)	74 (17)
CMS2	159 (31)	132 (31)
CMS3	83 (16)	68 (16)
CMS4	147 (28)	120 (28)
NA	45 (9)	36 (8)
